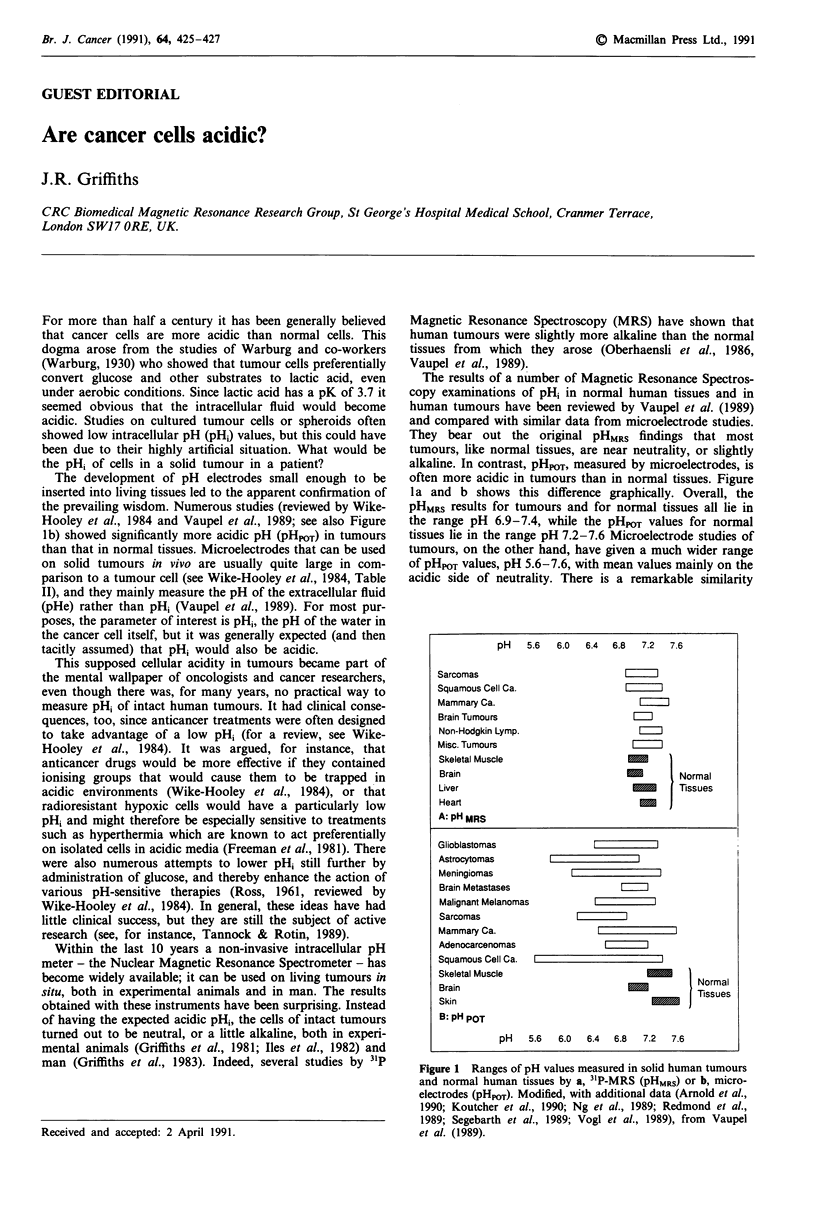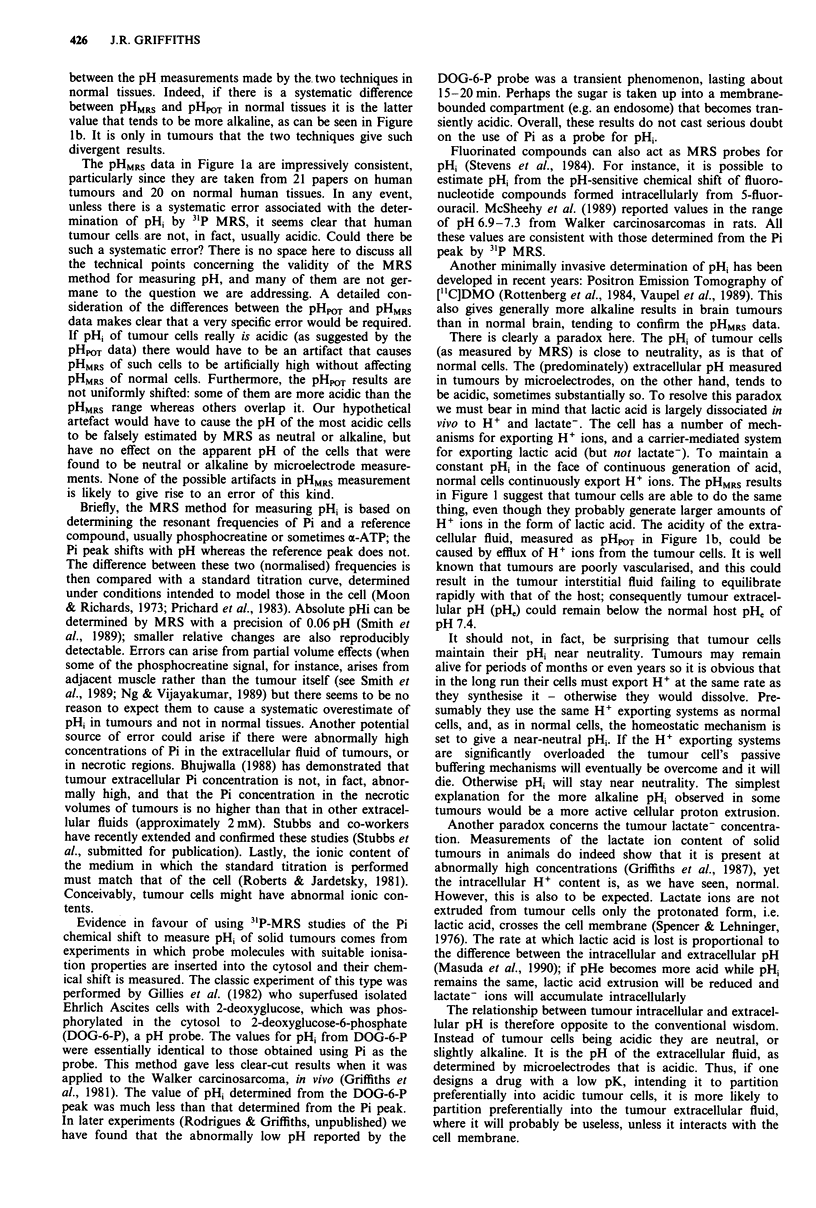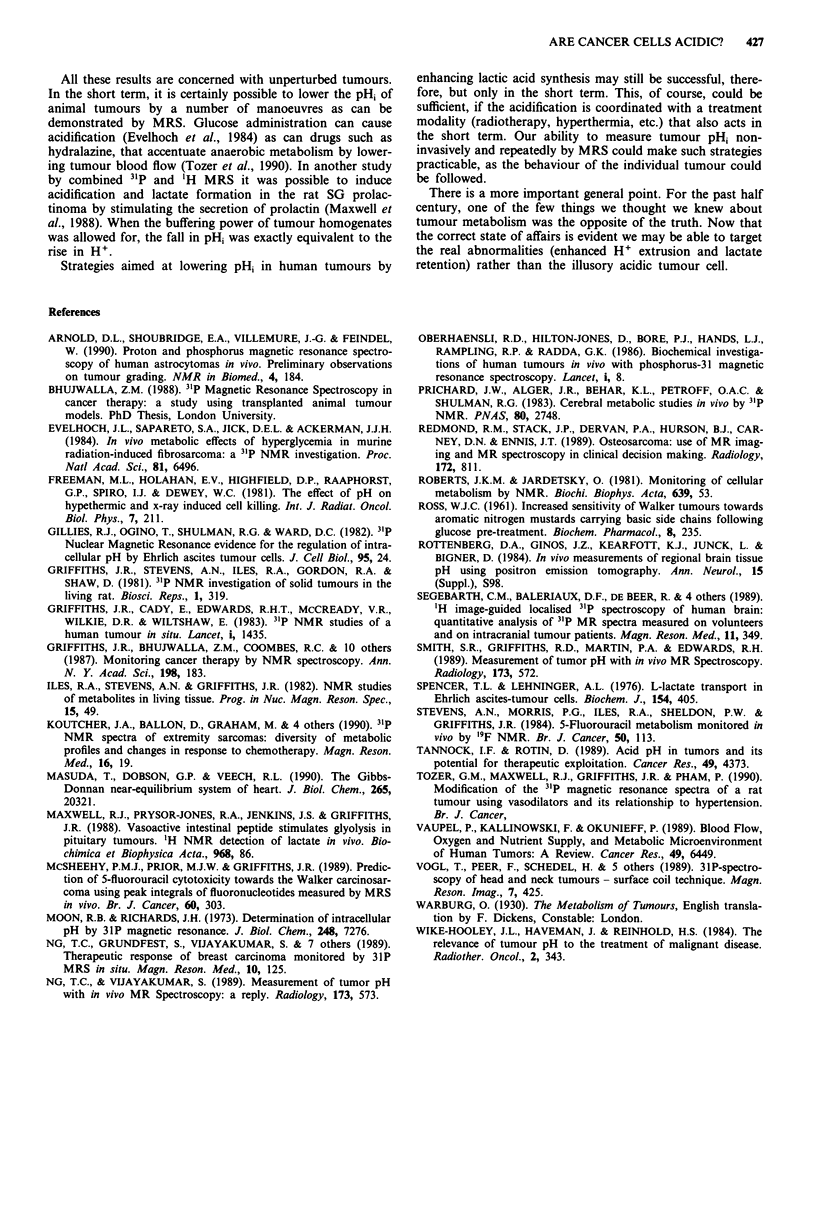# Are cancer cells acidic?

**DOI:** 10.1038/bjc.1991.326

**Published:** 1991-09

**Authors:** J. R. Griffiths


Br. J. Cancer (1991), 64, 425-427 © Macmillan Press Ltd., 1991

GUEST EDITORIAL

### Are cancer cells acidic?

J.R. Griffiths

*CRC Biomedical Magnetic Resonance Research Group, St George's Hospital Medical School, Cranmer Terrace, London SW17 0RE, UK.*

For more than half a century it has been generally believed that cancer cells are more acidic than normal cells. This dogma arose from the studies of Warburg and co-workers (Warburg, 1930) who showed that tumour cells preferentially convert glucose and other substrates to lactic acid, even under aerobic conditions. Since lactic acid has a pK of 3.7 it seemed obvious that the intracellular fluid would become acidic. Studies on cultured tumour cells or spheroids often showed low intracellular pH ($pH_i$) values, but this could have been due to their highly artificial situation. What would be the $pH_i$ of cells in a solid tumour in a patient?

The development of pH electrodes small enough to be inserted into living tissues led to the apparent confirmation of the prevailing wisdom. Numerous studies (reviewed by Wike-Hooley *et al.*, 1984 and Vaupel *et al.*, 1989; see also Figure 1b) showed significantly more acidic pH ($pH_{POT}$) in tumours than that in normal tissues. Microelectrodes that can be used on solid tumours *in vivo* are usually quite large in comparison to a tumour cell (see Wike-Hooley *et al.*, 1984, Table II), and they mainly measure the pH of the extracellular fluid (pHe) rather than $pH_i$ (Vaupel *et al.*, 1989). For most purposes, the parameter of interest is $pH_i$, the pH of the water in the cancer cell itself, but it was generally expected (and then tacitly assumed) that $pH_i$ would also be acidic.

This supposed cellular acidity in tumours became part of the mental wallpaper of oncologists and cancer researchers, even though there was, for many years, no practical way to measure $pH_i$ of intact human tumours. It had clinical consequences, too, since anticancer treatments were often designed to take advantage of a low $pH_i$ (for a review, see Wike-Hooley *et al.*, 1984). It was argued, for instance, that anticancer drugs would be more effective if they contained ionising groups that would cause them to be trapped in acidic environments (Wike-Hooley *et al.*, 1984), or that radioresistant hypoxic cells would have a particularly low $pH_i$ and might therefore be especially sensitive to treatments such as hyperthermia which are known to act preferentially on isolated cells in acidic media (Freeman *et al.*, 1981). There were also numerous attempts to lower $pH_i$ still further by administration of glucose, and thereby enhance the action of various pH-sensitive therapies (Ross, 1961, reviewed by Wike-Hooley *et al.*, 1984). In general, these ideas have had little clinical success, but they are still the subject of active research (see, for instance, Tannock & Rotin, 1989).

Within the last 10 years a non-invasive intracellular pH meter – the Nuclear Magnetic Resonance Spectrometer – has become widely available; it can be used on living tumours *in situ*, both in experimental animals and in man. The results obtained with these instruments have been surprising. Instead of having the expected acidic $pH_i$, the cells of intact tumours turned out to be neutral, or a little alkaline, both in experimental animals (Griffiths *et al.*, 1981; Iles *et al.*, 1982) and man (Griffiths *et al.*, 1983). Indeed, several studies by $^{31}P$ Magnetic Resonance Spectroscopy (MRS) have shown that human tumours were slightly more alkaline than the normal tissues from which they arose (Oberhaensli *et al.*, 1986, Vaupel *et al.*, 1989).

The results of a number of Magnetic Resonance Spectroscopy examinations of $pH_i$ in normal human tissues and in human tumours have been reviewed by Vaupel *et al.* (1989) and compared with similar data from microelectrode studies. They bear out the original $pH_{MRS}$ findings that most tumours, like normal tissues, are near neutrality, or slightly alkaline. In contrast, $pH_{POT}$, measured by microelectrodes, is often more acidic in tumours than in normal tissues. Figure 1a and b shows this difference graphically. Overall, the $pH_{MRS}$ results for tumours and for normal tissues all lie in the range pH 6.9–7.4, while the $pH_{POT}$ values for normal tissues lie in the range pH 7.2–7.6 Microelectrode studies of tumours, on the other hand, have given a much wider range of $pH_{POT}$ values, pH 5.6–7.6, with mean values mainly on the acidic side of neutrality. There is a remarkable similarity

**Figure 1** Ranges of pH values measured in solid human tumours and normal human tissues by **a**, $^{31}P$-MRS ($pH_{MRS}$) or **b**, microelectrodes ($pH_{POT}$). Modified, with additional data (Arnold *et al.*, 1990; Koutcher *et al.*, 1990; Ng *et al.*, 1989; Redmond *et al.*, 1989; Segebarth *et al.*, 1989; Vogl *et al.*, 1989), from Vaupel *et al.* (1989).

Received and accepted: 2 April 1991.

between the pH measurements made by the two techniques in normal tissues. Indeed, if there is a systematic difference between $pH_{MRS}$ and $pH_{POT}$ in normal tissues it is the latter value that tends to be more alkaline, as can be seen in Figure 1b. It is only in tumours that the two techniques give such divergent results.

The $pH_{MRS}$ data in Figure 1a are impressively consistent, particularly since they are taken from 21 papers on human tumours and 20 on normal human tissues. In any event, unless there is a systematic error associated with the determination of $pH_i$ by $^{31}P$ MRS, it seems clear that human tumour cells are not, in fact, usually acidic. Could there be such a systematic error? There is no space here to discuss all the technical points concerning the validity of the MRS method for measuring pH, and many of them are not germane to the question we are addressing. A detailed consideration of the differences between the $pH_{POT}$ and $pH_{MRS}$ data makes clear that a very specific error would be required. If $pH_i$ of tumour cells really *is* acidic (as suggested by the $pH_{POT}$ data) there would have to be an artifact that causes $pH_{MRS}$ of such cells to be artificially high without affecting $pH_{MRS}$ of normal cells. Furthermore, the $pH_{POT}$ results are not uniformly shifted: some of them are more acidic than the $pH_{MRS}$ range whereas others overlap it. Our hypothetical artefact would have to cause the pH of the most acidic cells to be falsely estimated by MRS as neutral or alkaline, but have no effect on the apparent pH of the cells that were found to be neutral or alkaline by microelectrode measurements. None of the possible artifacts in $pH_{MRS}$ measurement is likely to give rise to an error of this kind.

Briefly, the MRS method for measuring $pH_i$ is based on determining the resonant frequencies of Pi and a reference compound, usually phosphocreatine or sometimes α-ATP; the Pi peak shifts with pH whereas the reference peak does not. The difference between these two (normalised) frequencies is then compared with a standard titration curve, determined under conditions intended to model those in the cell (Moon & Richards, 1973; Prichard *et al.*, 1983). Absolute pHi can be determined by MRS with a precision of 0.06 pH (Smith *et al.*, 1989); smaller relative changes are also reproducibly detectable. Errors can arise from partial volume effects (when some of the phosphocreatine signal, for instance, arises from adjacent muscle rather than the tumour itself (see Smith *et al.*, 1989; Ng & Vijayakumar, 1989) but there seems to be no reason to expect them to cause a systematic overestimate of $pH_i$ in tumours and not in normal tissues. Another potential source of error could arise if there were abnormally high concentrations of Pi in the extracellular fluid of tumours, or in necrotic regions. Bhujwalla (1988) has demonstrated that tumour extracellular Pi concentration is not, in fact, abnormally high, and that the Pi concentration in the necrotic volumes of tumours is no higher than that in other extracellular fluids (approximately 2 mM). Stubbs and co-workers have recently extended and confirmed these studies (Stubbs *et al.*, submitted for publication). Lastly, the ionic content of the medium in which the standard titration is performed must match that of the cell (Roberts & Jardetsky, 1981). Conceivably, tumour cells might have abnormal ionic contents.

Evidence in favour of using $^{31}P$-MRS studies of the Pi chemical shift to measure $pH_i$ of solid tumours comes from experiments in which probe molecules with suitable ionisation properties are inserted into the cytosol and their chemical shift is measured. The classic experiment of this type was performed by Gillies *et al.* (1982) who superfused isolated Ehrlich Ascites cells with 2-deoxyglucose, which was phosphorylated in the cytosol to 2-deoxyglucose-6-phosphate (DOG-6-P), a pH probe. The values for $pH_i$ from DOG-6-P were essentially identical to those obtained using Pi as the probe. This method gave less clear-cut results when it was applied to the Walker carcinosarcoma, *in vivo* (Griffiths *et al.*, 1981). The value of $pH_i$ determined from the DOG-6-P peak was much less than that determined from the Pi peak. In later experiments (Rodrigues & Griffiths, unpublished) we have found that the abnormally low pH reported by the DOG-6-P probe was a transient phenomenon, lasting about 15–20 min. Perhaps the sugar is taken up into a membrane-bounded compartment (e.g. an endosome) that becomes transiently acidic. Overall, these results do not cast serious doubt on the use of Pi as a probe for $pH_i$.

Fluorinated compounds can also act as MRS probes for $pH_i$ (Stevens *et al.*, 1984). For instance, it is possible to estimate $pH_i$ from the pH-sensitive chemical shift of fluoronucleotide compounds formed intracellularly from 5-fluorouracil. McSheehy *et al.* (1989) reported values in the range of pH 6.9–7.3 from Walker carcinosarcomas in rats. All these values are consistent with those determined from the Pi peak by $^{31}P$ MRS.

Another minimally invasive determination of $pH_i$ has been developed in recent years: Positron Emission Tomography of [$^{11}C$]DMO (Rottenberg *et al.*, 1984, Vaupel *et al.*, 1989). This also gives generally more alkaline results in brain tumours than in normal brain, tending to confirm the $pH_{MRS}$ data.

There is clearly a paradox here. The $pH_i$ of tumour cells (as measured by MRS) is close to neutrality, as is that of normal cells. The (predominately) extracellular pH measured in tumours by microelectrodes, on the other hand, tends to be acidic, sometimes substantially so. To resolve this paradox we must bear in mind that lactic acid is largely dissociated *in vivo* to $H^+$ and lactate$^-$. The cell has a number of mechanisms for exporting $H^+$ ions, and a carrier-mediated system for exporting lactic acid (but *not* lactate$^-$). To maintain a constant $pH_i$ in the face of continuous generation of acid, normal cells continuously export $H^+$ ions. The $pH_{MRS}$ results in Figure 1 suggest that tumour cells are able to do the same thing, even though they probably generate larger amounts of $H^+$ ions in the form of lactic acid. The acidity of the extracellular fluid, measured as $pH_{POT}$ in Figure 1b, could be caused by efflux of $H^+$ ions from the tumour cells. It is well known that tumours are poorly vascularised, and this could result in the tumour interstitial fluid failing to equilibrate rapidly with that of the host; consequently tumour extracellular pH ($pH_e$) could remain below the normal host $pH_e$ of pH 7.4.

It should not, in fact, be surprising that tumour cells maintain their $pH_i$ near neutrality. Tumours may remain alive for periods of months or even years so it is obvious that in the long run their cells must export $H^+$ at the same rate as they synthesise it – otherwise they would dissolve. Presumably they use the same $H^+$ exporting systems as normal cells, and, as in normal cells, the homeostatic mechanism is set to give a near-neutral $pH_i$. If the $H^+$ exporting systems are significantly overloaded the tumour cell's passive buffering mechanisms will eventually be overcome and it will die. Otherwise $pH_i$ will stay near neutrality. The simplest explanation for the more alkaline $pH_i$ observed in some tumours would be a more active cellular proton extrusion.

Another paradox concerns the tumour lactate$^-$ concentration. Measurements of the lactate ion content of solid tumours in animals do indeed show that it is present at abnormally high concentrations (Griffiths *et al.*, 1987), yet the intracellular $H^+$ content is, as we have seen, normal. However, this is also to be expected. Lactate ions are not extruded from tumour cells only the protonated form, i.e. lactic acid, crosses the cell membrane (Spencer & Lehninger, 1976). The rate at which lactic acid is lost is proportional to the difference between the intracellular and extracellular pH (Masuda *et al.*, 1990); if pHe becomes more acid while $pH_i$ remains the same, lactic acid extrusion will be reduced and lactate$^-$ ions will accumulate intracellularly

The relationship between tumour intracellular and extracellular pH is therefore opposite to the conventional wisdom. Instead of tumour cells being acidic they are neutral, or slightly alkaline. It is the pH of the extracellular fluid, as determined by microelectrodes that is acidic. Thus, if one designs a drug with a low pK, intending it to partition preferentially into acidic tumour cells, it is more likely to partition preferentially into the tumour extracellular fluid, where it will probably be useless, unless it interacts with the cell membrane.

All these results are concerned with unperturbed tumours. In the short term, it is certainly possible to lower the $pH_i$ of animal tumours by a number of manoeuvres as can be demonstrated by MRS. Glucose administration can cause acidification (Evelhoch *et al.*, 1984) as can drugs such as hydralazine, that accentuate anaerobic metabolism by lowering tumour blood flow (Tozer *et al.*, 1990). In another study by combined $^{31}P$ and $^{1}H$ MRS it was possible to induce acidification and lactate formation in the rat SG prolactinoma by stimulating the secretion of prolactin (Maxwell *et al.*, 1988). When the buffering power of tumour homogenates was allowed for, the fall in $pH_i$ was exactly equivalent to the rise in $H^+$.

Strategies aimed at lowering $pH_i$ in human tumours by enhancing lactic acid synthesis may still be successful, therefore, but only in the short term. This, of course, could be sufficient, if the acidification is coordinated with a treatment modality (radiotherapy, hyperthermia, etc.) that also acts in the short term. Our ability to measure tumour $pH_i$ non-invasively and repeatedly by MRS could make such strategies practicable, as the behaviour of the individual tumour could be followed.

There is a more important general point. For the past half century, one of the few things we thought we knew about tumour metabolism was the opposite of the truth. Now that the correct state of affairs is evident we may be able to target the real abnormalities (enhanced $H^+$ extrusion and lactate retention) rather than the illusory acidic tumour cell.

### References

ARNOLD, D.L., SHOUBRIDGE, E.A., VILLEMURE, J.-G. & FEINDEL, W. (1990). Proton and phosphorus magnetic resonance spectroscopy of human astrocytomas *in vivo*. Preliminary observations on tumour grading. *NMR in Biomed.*, **4**, 184.

BHUJWALLA, Z.M. (1988). $^{31}P$ Magnetic Resonance Spectroscopy in cancer therapy: a study using transplanted animal tumour models. PhD Thesis, London University.

EVELHOCH, J.L., SAPARETO, S.A., JICK, D.E.L. & ACKERMAN, J.J.H. (1984). *In vivo* metabolic effects of hyperglycemia in murine radiation-induced fibrosarcoma: a $^{31}P$ NMR investigation. *Proc. Natl Acad. Sci.*, **81**, 6496.

FREEMAN, M.L., HOLAHAN, E.V., HIGHFIELD, D.P., RAAPHORST, G.P., SPIRO, I.J. & DEWEY, W.C. (1981). The effect of pH on hypethermic and x-ray induced cell killing. *Int. J. Radiat. Oncol. Biol. Phys.*, **7**, 211.

GILLIES, R.J., OGINO, T., SHULMAN, R.G. & WARD, D.C. (1982). $^{31}P$ Nuclear Magnetic Resonance evidence for the regulation of intracellular pH by Ehrlich ascites tumour cells. *J. Cell Biol.*, **95**, 24.

GRIFFITHS, J.R., STEVENS, A.N., ILES, R.A., GORDON, R.A. & SHAW, D. (1981). $^{31}P$ NMR investigation of solid tumours in the living rat. *Biosci. Reps.*, **1**, 319.

GRIFFITHS, J.R., CADY, E., EDWARDS, R.H.T., McCREADY, V.R., WILKIE, D.R. & WILTSHAW, E. (1983). $^{31}P$ NMR studies of a human tumour *in situ. Lancet*, **i**, 1435.

GRIFFITHS, J.R., BHUJWALLA, Z.M., COOMBES, R.C. & 10 others (1987). Monitoring cancer therapy by NMR spectroscopy. *Ann. N. Y. Acad. Sci.*, **198**, 183.

ILES, R.A., STEVENS, A.N. & GRIFFITHS, J.R. (1982). NMR studies of metabolites in living tissue. *Prog. in Nuc. Magn. Reson. Spec.*, **15**, 49.

KOUTCHER, J.A., BALLON, D., GRAHAM, M. & 4 others (1990). $^{31}P$ NMR spectra of extremity sarcomas: diversity of metabolic profiles and changes in response to chemotherapy. *Magn. Reson. Med.*, **16**, 19.

MASUDA, T., DOBSON, G.P. & VEECH, R.L. (1990). The Gibbs-Donnan near-equilibrium system of heart. *J. Biol. Chem.*, **265**, 20321.

MAXWELL, R.J., PRYSOR-JONES, R.A., JENKINS, J.S. & GRIFFITHS, J.R. (1988). Vasoactive intestinal peptide stimulates glyolysis in pituitary tumours. $^{1}H$ NMR detection of lactate *in vivo*. *Biochimica et Biophysica Acta.*, **968**, 86.

McSHEEHY, P.M.J., PRIOR, M.J.W. & GRIFFITHS, J.R. (1989). Prediction of 5-fluorouracil cytotoxicity towards the Walker carcinosarcoma using peak integrals of fluoronucleotides measured by MRS *in vivo*. *Br. J. Cancer*, **60**, 303.

MOON, R.B. & RICHARDS, J.H. (1973). Determination of intracellular pH by 31P magnetic resonance. *J. Biol. Chem.*, **248**, 7276.

NG, T.C., GRUNDFEST, S., VIJAYAKUMAR, S. & 7 others (1989). Therapeutic response of breast carcinoma monitored by 31P MRS *in situ*. *Magn. Reson. Med.*, **10**, 125.

NG, T.C., & VIJAYAKUMAR, S. (1989). Measurement of tumor pH with *in vivo* MR Spectroscopy: a reply. *Radiology*, **173**, 573.

OBERHAENSLI, R.D., HILTON-JONES, D., BORE, P.J., HANDS, L.J., RAMPLING, R.P. & RADDA, G.K. (1986). Biochemical investigations of human tumours *in vivo* with phosphorus-31 magnetic resonance spectroscopy. *Lancet*, **i**, 8.

PRICHARD, J.W., ALGER, J.R., BEHAR, K.L., PETROFF, O.A.C. & SHULMAN, R.G. (1983). Cerebral metabolic studies *in vivo* by $^{31}P$ NMR. *PNAS*, **80**, 2748.

REDMOND, R.M., STACK, J.P., DERVAN, P.A., HURSON, B.J., CARNEY, D.N. & ENNIS, J.T. (1989). Osteosarcoma: use of MR imaging and MR spectroscopy in clinical decision making. *Radiology*, **172**, 811.

ROBERTS, J.K.M. & JARDETSKY, O. (1981). Monitoring of cellular metabolism by NMR. *Biochi. Biophys. Acta*, **639**, 53.

ROSS, W.J.C. (1961). Increased sensitivity of Walker tumours towards aromatic nitrogen mustards carrying basic side chains following glucose pre-treatment. *Biochem. Pharmacol.*, **8**, 235.

ROTTENBERG, D.A., GINOS, J.Z., KEARFOTT, K.J., JUNCK, L. & BIGNER, D. (1984). *In vivo* measurements of regional brain tissue pH using positron emission tomography. *Ann. Neurol.*, **15** (Suppl.), S98.

SEGEBARTH, C.M., BALERIAUX, D.F., DE BEER, R. & 4 others (1989). $^{1}H$ image-guided localised $^{31}P$ spectroscopy of human brain: quantitative analysis of $^{31}P$ MR spectra measured on volunteers and on intracranial tumour patients. *Magn. Reson. Med.*, **11**, 349.

SMITH, S.R., GRIFFITHS, R.D., MARTIN, P.A. & EDWARDS, R.H. (1989). Measurement of tumor pH with *in vivo* MR Spectroscopy. *Radiology*, **173**, 572.

SPENCER, T.L. & LEHNINGER, A.L. (1976). L-lactate transport in Ehrlich ascites-tumour cells. *Biochem. J.*, **154**, 405.

STEVENS, A.N., MORRIS, P.G., ILES, R.A., SHELDON, P.W. & GRIFFITHS, J.R. (1984). 5-Fluorouracil metabolism monitored *in vivo* by $^{19}F$ NMR. *Br. J. Cancer*, **50**, 113.

TANNOCK, I.F. & ROTIN, D. (1989). Acid pH in tumors and its potential for therapeutic exploitation. *Cancer Res.*, **49**, 4373.

TOZER, G.M., MAXWELL, R.J., GRIFFITHS, J.R. & PHAM, P. (1990). Modification of the $^{31}P$ magnetic resonance spectra of a rat tumour using vasodilators and its relationship to hypertension. *Br. J. Cancer*,

VAUPEL, P., KALLINOWSKI, F. & OKUNIEFF, P. (1989). Blood Flow, Oxygen and Nutrient Supply, and Metabolic Microenvironment of Human Tumors: A Review. *Cancer Res.*, **49**, 6449.

VOGL, T., PEER, F., SCHEDEL, H. & 5 others (1989). 31P-spectroscopy of head and neck tumours – surface coil technique. *Magn. Reson. Imag.*, **7**, 425.

WARBURG, O. (1930). *The Metabolism of Tumours*, English translation by F. Dickens, Constable: London.

WIKE-HOOLEY, J.L., HAVEMAN, J. & REINHOLD, H.S. (1984). The relevance of tumour pH to the treatment of malignant disease. *Radiother. Oncol.*, **2**, 343.